# Zelquistinel Is an Orally Bioavailable Novel NMDA Receptor Allosteric Modulator That Exhibits Rapid and Sustained Antidepressant-Like Effects

**DOI:** 10.1093/ijnp/pyac043

**Published:** 2022-07-26

**Authors:** Jeffrey S Burgdorf, Xiao-Lei Zhang, Patric K Stanton, Joseph R Moskal, John E Donello

**Affiliations:** Gate Neurosciences, Inc., Carmel, Indiana, USA; Department of Cell Biology and Anatomy, New York Medical College, Valhalla, New York, USA; Department of Cell Biology and Anatomy, New York Medical College, Valhalla, New York, USA; Falk Center for Molecular Therapeutics, Department of Biomedical Engineering, McCormick School of Engineering and Applied Sciences, Northwestern University, Evanston, Illinois, USA; Gate Neurosciences, Inc., Carmel, Indiana, USA

**Keywords:** Antidepressant, NMDA receptor, synaptic plasticity, depression, major depressive disorder, zelquistinel

## Abstract

**Background:**

The role of glutamatergic receptors in major depressive disorder continues to be of great interest for therapeutic development. Recent studies suggest that both negative and positive modulation of N-methyl-D-aspartate receptors (NMDAR) can produce rapid antidepressant effects. Here we report that zelquistinel, a novel NMDAR allosteric modulator, exhibits high oral bioavailability and dose-proportional exposures in plasma and the central nervous system and produces rapid and sustained antidepressant-like effects in rodents by enhancing activity-dependent, long-term synaptic plasticity.

**Methods:**

NMDAR-mediated functional activity was measured in cultured rat brain cortical neurons (calcium imaging), hNR2A or B subtype-expressing HEK cells, and synaptic plasticity in rat hippocampal and medial prefrontal cortex slices in vitro. Pharmacokinetics were evaluated in rats following oral administration. Antidepressant-like effects were assessed in the rat forced swim test and the chronic social deficit mouse model. Target engagement and the safety/tolerability profile was assessed using phencyclidine-induced hyperlocomotion and rotarod rodent models.

**Results:**

Following a single oral dose, zelquistinel (0.1–100 µg/kg) produced rapid and sustained antidepressant-like effects in the rodent depression models. Brain/ cerebrospinal fluid concentrations associated with zelquistinel antidepressant-like activity also increased NMDAR function and rapidly and persistently enhanced activity-dependent synaptic plasticity (long-term potentiation), suggesting that zelquistinel produces antidepressant-like effects by enhancing NMDAR function and synaptic plasticity. Furthermore, Zelquistinel inhibited phencyclidine (an NMDAR antagonist)-induced hyperlocomotion and did not impact rotarod performance.

**Conclusions:**

Zelquistinel produces rapid and sustained antidepressant effects by positively modulating the NMDARs, thereby enhancing long-term potentiation of synaptic transmission.

**Significance Statement**

Major depressive disorder (MDD) is a prevalent mental illness associated with significant personal, social, and economic burden. Nearly one-half of all patients with MDD have inadequate responses to antidepressants, which often take weeks to reach full effect and are associated with adverse events that can lead to discontinuation of treatment. New therapies with novel mechanisms of action and a rapid onset would be advantageous to patients with MDD. Modulation of N-methyl-D-aspartate receptors (NMDARs) can produce rapid and sustained clinically relevant antidepressant effects. Zelquistinel, a novel, orally bioavailable NMDAR allosteric modulator that binds to a unique site on NMDAR, is currently in phase 2 development for the treatment of MDD. We report here that orally administered zelquistinel exhibits a favorable safety/tolerability profile and can rapidly and persistently enhance activity-dependent long-term synaptic plasticity, accompanied by a rapid-acting and long-lasting antidepressant-like effects in rodents.

## Introduction

N-methyl-D-aspartate (NMDA) receptors (NMDAR) have been implicated in a number of physiologic and pathologic processes, including mood disorders (Ghasemi et al., 2014; Vasilescu et al., 2017), schizophrenia (Coyle, 2012; Goff, 2012), pain (Millecamps et al., 2007; Zhou et al., 2011), Rett syndrome (Patrizi et al., 2016), and cognitive decline due to aging (Burgdorf et al., 2011). Ketamine and other NMDAR antagonists have demonstrated rapid onset of antidepressant activity in patients with treatment-resistant depression (Berman et al., 2000; Zarate et al., 2006; Preskorn et al., 2008; Ibrahim et al., 2012); however, ketamine is associated with significant psychotomimetic, sedative, and ataxic side effects and memory defects, all of which have limited its therapeutic utility (Backonja et al., 1994; Niesters et al., 2014).

Ketamine’s antidepressant effects are postulated to be due to increased glutamatergic transmission and subsequent enhancement of synaptic plasticity (Li et al., 2010; Henley and Wilkinson, 2016; Zanos and Gould, 2018). Interestingly, NMDAR antagonists acutely inhibit glutamatergic neurotransmission and NMDAr-dependent synaptic plasticity. A set of mechanistic studies suggests that ketamine indirectly enhances glutamatergic neurotransmission by promoting GABAergic disinhibition via blockade of GABAergic NR2B receptors and a subsequent burst of extracellular glutamate (Gerhard et al., 2020). Another study posits ketamine’s effect is mediated by a metabolite of ketamine that directly promotes glutamatergic neurotransmission without requiring activity at NMDAR receptors (Zanos et al., 2016; Aleksandrova et al., 2017). Enhanced glutamatergic neurotransmission appears to be essential for ketamine’s antidepressant activity. Although the molecular mechanism by which ketamine provokes synaptic plasticity is not fully resolved, it is clear that NMDAR antagonism by ketamine elicits side effects that limit clinical utility.

Positive modulation of NMDARs represents a novel path to enhance synaptic plasticity and normalize mood without inducing adverse central nervous system (CNS) effects that often accompany NMDAR antagonism (Preskorn et al., 2015; Khan et al., 2018; Houck et al., 2019). Rapastinel is a tetrapeptide that exhibits a biphasic modulation of NMDARs. Intravenous rapastinel produces rapid and sustained antidepressant effects in rodents (Burgdorf et al., 2013; Yang et al., 2016) and in multiple clinical studies with depressed patients (Preskorn et al., 2015), although recent clinical trial experiences with rapastinel have been mixed. As a natural peptide, the drug-like properties of rapastinel were not optimized; the molecule exhibits a low partition into the central compartment and a short plasma half-life that necessitated intravenous administration. Recently Pothula et al. (2021) reported a new positive NMDAR modulator, zelquistinel (AGN-241751), which exhibits antidepressant-like activity via positive modulation of GluN2B containing NMDARs in mPFC excitatory neurons. Although zelquistinel and rapastinel share a mechanism of action, unlike rapastinel, zelquistinel is not a peptide and has improved drug-like properties that may enable oral administration.

The present studies were performed to characterize zelquistinel’s in vitro and in vivo pharmacology and determine its potential as a new investigative oral treatment for depression. Previous studies have shown that activity-dependent long-term synaptic plasticity is impaired in depression (Burgdorf et al., 2015). Therefore, we addressed three specific questions: (1) does zelquistinel positively modulate NMDAR via a similar mechanism as rapastinel? (2) does oral zelquistinel have sufficient CNS bioavailability to produce long-lasting metaplasticity actions via induction of long-term potential (LTP) at hippocampal and mPFC synapses? and (3) does the dose-response relation of zelquistinel modulation of NMDAR conductances, plasticity, and antidepressant-like actions indicate that it has suitable drug-like properties for an oral therapeutic for major depressive disorder?

## Materials and Methods

### Animals

Experiments were approved by the Allergan, New York Medical College, Northshore Hospital (Evanston, IL, USA) IACUC committees and were carried out in accordance with either the Guide for the Care and Use of Laboratory Animals as adopted and promulgated by the US National Institutes of Health or with the European Communities Council Directive (Directive 2010/63 EU into the Irish SI 543/2012).

### Drugs

Zelquistinel was synthesized in free-base form by Sai Life Sciences (Hyderabad, India). For the behavioral studies, zelquistinel was dissolved in 0.9% sterile saline and administered orally (PO).

### Radioligand Binding Assays for Off-Target Activity

Off-target activities were conducted by Cerep (www.cerep.fr) using their high-throughput profile of binding assays. Cerep’s high-throughput profile consists of a broad collection of 80 transmembrane and soluble receptors, ion channels, and monoamine transporters. Results showing an inhibition (or stimulation for assays run in basal conditions) >50% were considered to represent significant effects of the test compound. Results showing an inhibition (or stimulation) between 25% and 50% were indicative of weak to moderate effects. Results showing an inhibition (or stimulation) <25% were not considered significant and attributed to the variability of the signal at approximately the control level.

### Intracellular Ca^2+^ Influx Measurement

Rat brain cortical neurons were obtained from Life Technologies (A10840-02; Frederick, MD, USA). Cortical cells from embryonic day 18 were seeded onto coverslips coated with poly-D-lysine and cultured per the manufacturer’s instructions. The neurons were used 3 weeks after plating. Subtype activities were measured using HEK293 cells stably expressing human NR1-NR2A or NR1-NR2B receptors with tetracycline-inducible promoter. At 24 hours before recording, 2 µg/mL tetracycline and 1 mM ketamine were added to the culture medium.

Dye loading, optical recording, and data analysis of intracellular Ca^2+^ influx were performed according to previously published methods (Donello et al., 2019).

### Slice Electrophysiology

Coronal slices containing mPFC were prepared from 6- to 10-week-old Sprague Dawley male rats. Rats were deeply anesthetized with isoflurane and decapitated. Brains were removed rapidly and submerged in ice-cold slice cutting solution (approximately 0–2°C), which contained (in mM) 12.5 NaCl, 2.5 KCl, 0.5 CaCl_2_, 4 MgCl_2_, 1.25 NaH_2_PO_4_, 26 NaHCO_3_, 10 glucose, and 200 sucrose at pH 7.4 and gassed continuously with 95% O^2^/5% CO_2_. Modified coronal slices containing prelimbic (PrL) and infralimbic regions were cut at 300-µm thickness using a Vibratome (Leica VT1200S) according to the method of Parent et al. (2010) and transferred to an interface holding chamber containing oxygenated (95% O_2_/5% CO_2_) cutting solution for incubation at room temperature for 30 minutes. These were then transferred to oxygenated artificial cerebrospinal fluid (aCSF), which contained in (mM) 125 NaCl, 2.5 KCl, 2 CaCl_2_, 1 MgCl_2_, 1.25 NaH_2_PO_4_, 26 NaHCO_3_, and 10 dextrose for another 30 minutes before recording. For extracellular field EPSP (fEPSP) recording, slices were transferred to a Haas-style interface recording chamber continuously perfused at 3 mL/min with oxygenated ACSF at 32°C ± 0.5°C. For intracellular recording, slices were placed in a submerged recording chamber (RC-27L, Warner Instruments, Hamden, CT, USA) and held down with a nylon grid. For all recordings, the bath solution (aCSF) contained picrotoxin (20 µM), and CGP 55845 hydrochloride (200 nM) was present throughout the experiments to block γ-aminobutyric acid (GABA) type A (GABA_A_) and type B (GABA_B_) receptors, respectively.

Whole-cell recordings in mPFC neurons, excitatory postsynaptic currents (EPSCs) recordings, and fEPSP/LTP recordings were performed as previously described (Donello et al., 2019).

For extracellular recordings, low-resistance recording electrodes were made from thin-walled borosilicate glass (1–2 MΩ after filling with aCSF) and inserted into layers 3–4 of the PrL region of the mPFC to record fEPSPs. A bipolar stainless-steel stimulating electrode (FHC Co., Bowdoin, ME, USA) was placed on mixed efferent input pathways that included hippocampal inputs in mPFC close to the recording electrode, and constant current stimulus intensity adjusted to evoke approximately half-maximal fEPSPs was given once each 30 seconds (50–100 µA; 100-microsecond duration). fEPSP slope was measured before and after induction of LTP by linear interpolation from 20% to 80% of maximum negative deflection, and slopes were confirmed to be stable to within ±10% for at least 15 minutes before commencing an experiment. LTP was induced by stimulation of the mixed efferent pathway with 3 high-frequency theta burst stimulus trains of 10 × 100-Hz/5 pulse bursts each applied at an inter-burst interval of 200 milliseconds. Each train was 2 seconds in duration, and trains were applied 3 minutes apart. Signals were recorded using a differential AC amplifier (A-M Systems, Model 1700, Sequim, WA, USA) and digitized with an A/D board (1608GX-2AO) from DataWave Technologies. All recording and analysis were controlled by SciWorks (DataWave v9.1).

Patch pipettes were pulled from borosilicate glass (1B150F-4, World Precision Instruments) using a Flaming/Brown micropipette puller (P-97, Sutter Instruments). The composition of the patch pipette solution for NMDA current recordings was (in mM) 135 mM CsMeSO_3_, 8 mM NaCl, 10 HEPES, 2 Mg-adenosine 5′-triphosphate, 0.3 Na-guanine triphosphate, 0.5 ethylene glycol-bis(β-aminoethyl ether)-N,N,N′,N′-tetraacetic acid, 1 QX-314. The patch pipette solution pH was adjusted to 7.25 with CsOH and had an osmolarity of 280 ± 10 mOsm. When filled with this solution, patch pipettes had tip resistances of 5–6 MΩ.

The submerged recording chamber was mounted on a Zeiss Axioskop 2 FS upright microscope equipped with infrared differential interference contrast optics. After transfer to the recording chamber, slices were continually perfused with oxygenated modified aCSF. Pyramidal neurons in layers II–III of the PrL region of the medial prefrontal cortex were visualized with a 63× water immersion lens and patched under voltage-clamp configuration. EPSCs were recorded using a MultiClamp 700B (Axon Instruments, Foster City, CA, USA) with the low-pass filter setting at 1–3 kHz, series resistance was compensated in the voltage-clamp mode during the recordings period, and patched cells whose series resistance changed by >10% were rejected. NMDAR-mediated synaptic currents were pharmacologically isolated with aCSF that contained 3 mM CaCl_2_, zero added Mg^2+^, and working concentrations of α-amino-3-hydroxy-5-methyl-4-isoxazolepropionic acid, GABA_A_, and GABA_B_ receptor blockers and by clamping the membrane resting potential at −40 mV to remove residual Mg^2+^ block from nanomolar levels of Mg^2+^ in the aCSF. Data were acquired with a 32-bit D/A interface (Digidata 1550, Axon Instruments) stored on a PC-compatible computer and analyzed using PCLAMP software (v9, Axon Instruments). MiniEPSCs were automatically analyzed with Minianalysis (V6.0.3, Synaptosoft).

### Pharmacokinetic Study

Male Sprague Dawley rats were divided into 2 groups: Group 1 (2 mg/kg, IV; n = 3) and Group 2 (10 mg/kg, PO; n = 3). Blood samples (approximately 120 µL) were collected from freely moving jugular vein cannulated rats such that samples were obtained at pre-dose; 0.08, 0.25, 0.5, 1, 2, 4, 8, and 24 hours post-IV dose and pre-dose; and 0.25, 0.5, 1, 2, 4, 6, 8, and 24 hours post-PO dose. Immediately after blood collection, plasma was harvested by centrifugation and stored at −70ºC until analysis. At Covance Laboratories Inc, 24 male Sprague Dawley rats were divided into 2 groups: Group 1 (100 µg/kg, PO; n = 12) and Group 2 (3 µg/kg, PO; n = 12). Blood (approximately 0.5 mL) was collected from a jugular vein via syringe and needle and transferred into tubes containing K2-EDTA anticoagulant from 3 animals per group at time points of 0.5, 1, 2, 4, 8, 10, 12, and 24 hours post-dose. Blood was maintained on wet ice prior to centrifugation to obtain plasma. Centrifugation began within 1 hour of collection. Plasma was placed on dry ice prior to storage at approximately −70ºC. Following blood collection, animals were killed by overdose of isoflurane anesthesia. At that time, CSF was collected from the cisterna magna according to Covance’s standard operating procedures. Samples were stored on dry ice prior to storage at approximately −70°C. Following CSF collection, animals were exsanguinated via whole-body perfusion with heparinized, sodium nitrite saline. The brain was excised, rinsed with saline, blotted dry, weighed, and placed on dry ice prior to storage at approximately −70°C. Noncompartmental analysis module in Phoenix WinNonlin (Version 6.3) was used to assess the pharmacokinetic parameters.

### Animals and Dosing of Zelquistinel for Long-term Metaplasticity of LTP

Male, 2- to 3-month-old Sprague Dawley rats (Charles River Laboratories, Kingston, NY, USA) were used. Rats were housed in Lucite cages with wood chip bedding, maintained on a 12:12 light:dark cycle, and given ad libitum access to Purina LabDiet (St. Louis, MO, USA) and tap water throughout the study. Zelquistinel (10 ng/kg to 10 mg/kg) were administered by gastric gavage. Vehicle controls were run in parallel with each dose and data were pooled over all experiments. At 24 hours, 72 hours, 1 week, 2 weeks, or 4 weeks after oral dosing, slices were prepared for measurement of LTP at Schaffer collateral-CA1 synapses in the hippocampus or at mixed excitatory synapses in layer II/III in the mPFC using slice electrophysiology methods described above.

### Forced Swim Test (FST)

The Porsolt FST was adapted for use in rats as previously described (Burgdorf et al., 2015; Donello et al., 2019). Rats were habituated to the test apparatus (cylindrical tube [46 cm tall × 20 cm diameter] filled to 30 cm with room temperature tap water) for 15 minutes on the day before testing. On subsequent days, animals were administrated zelquistinel (PO) or vehicle (sterile saline [1 mL/kg], PO). The time spent immobile during a 5-minute period was measured at 60 minutes post dosing. The same animals were then retested at 24 hours, 7 days, and 14 days post dose, and potential sex differences were also examined at the optimal dose identified (30 µg/kg, PO) at each of these timepoints.

### Chronic Social Defeat (CSD) Mouse Model

CSD was used to induce a depressive-like state in mice (Charles River, UK) at Transpharmation Ireland Ltd (Ireland) using a protocol for aggressor mice screening, CSD, and social preference (SP) test adapted from Golden et al. (2011) as the measure of social approach behavior. We chose to perform these tests in male mice given that the SP test is best validated in male mice (i.e., Golden et al., 2011); however, recent studies have shown that social defeat can also be performed in female mice (Takahashi et al., 2017; Newman et al., 2019).

The SP score (as expressed as a percentage) was calculated by dividing the time spent in the “interaction zone” when the target aggressor was present by the time spent in the same zone when the target was absent.

CD-1 male mice were subjected to aggression screening. Briefly, the screener mouse was placed into the home cage of an aggressor for a maximum of 180 seconds. After noting the latency of the aggressor to first attack, the screener mouse was removed from the aggressor and placed in its home cage. In total, 3 screening sessions were performed, 1 session per day for 3 consecutive days. CD-1 mice had to attack in at least 2 consecutive screening sessions, and the latency for initial attack must be <90 seconds.

C57BL/6J male mice were submitted to 10 days of CSD. Briefly, each selected CD-1 aggressor mouse was placed in one-half of a cage divided by custom-made separator 24 hours prior to the start and after the end of each defeat session. During each session of direct physical interaction, which was 10 minutes long and occurred daily for 10 consecutive days, CD-1 mice would initiate multiple confrontations. After the first sign of defeat posture, any further attacks from the CD-1 aggressor were terminated. Mice were housed within their respective area of the cage for the next 24 hours. For each subsequent daily defeat, the intruder animals were exposed to a novel CD-1 mouse to prevent any habituation to the resident aggressor. Control animals were placed in pairs within an identical home cage setup, 1 control animal per side. They were rotated to a new cage daily, but they never experienced direct physical contact with their cage mates.

After CSD, baseline SP was assessed in the first SP test, and the mice were divided into stress-resilient and stress-susceptible groups. These mice were then randomized to receive zelquistinel (30 μg/kg to 1 mg/kg, PO) or s.c. injection of 10 mg/kg ketamine. Antidepressant-like effect was tested in the SP test 24 hours after administration of ketamine or 1 hour after administration of zelquistinel.

### Phencyclidine (PCP)-Induced Hyperlocomotion

Studies were conducted at Amylgen SAS (Montpellier, France). PCP (2.5 mg/kg, s.c.)-induced hyperlocomotion was recorded in the open field (OF) activity test. The OF was made of opaque blue plexiglass. The arena is composed of a large square box (50 cm × 50 cm × 50 cm). Animal movements were followed by an overhead camera located above the apparatus. Each rat was placed in the center of the arena and allowed to move freely through the arena during the single 70-minute session. The horizontal locomotor activity was assessed by counting the total distance traveled measured in centimeters within 5-minute time intervals for the whole session time (Sun et al., 2009; Maple et al., 2017); the data were plotted as activity vs time.

### Rotarod

Rotarod testing was conducted as described (Nadeson et al., 2002) using a 4-station rotarod apparatus (Med Associates, USA). One day before testing, animals (male 2- to 3-month-old Sprague Dawley rats) received 3 rotarod habituation sessions with at least 30 minutes between each session and an additional habituation session immediately before dosing (0 minutes). Animals were tested 5, 30, 60, and 120 minutes after dosing using a within-subject design. Habituation and testing consisted of placing rats onto the fixed speed version of the rotarod test (16 RPM) for 300 seconds; the latency to fall off the rotarod was recorded. Potential sex differences were also examined at the optimal dose identified in the FST (30 µg/kg, PO) along with a positive control (Ifenprodil) in female rats.

### Statistical Analysis

Data from calcium imaging studies were analyzed using paired *t* test (Excel) comparing fluorescence before and after glutamate application. Effects of multiple concentrations of zelquistinel on pharmacologically isolated NMDAR-mediated EPSCs in mPFC pyramidal neurons were assessed by 1-way ANOVA with Bonferroni correction for multiple comparisons comparing pre-drug baselines to EPSC amplitude following 20 minutes of drug bath application.

Since equal variance could not be assumed for differing drug concentrations, magnitude of LTP in acute drug-treated mPFC slices 47–50 minutes post-TBS were evaluated by Kruskal-Wallis 1-way ANOVA, followed by Kruskal-Wallis multiple comparisons Z-values test, comparing each concentration to the magnitude of LTP in untreated control slices.

For metaplasticity experiments where LTP was evaluated in vitro 24 hours to 4 weeks post application for multiple drug concentrations administered in vivo, 1-way ANOVA followed by Dunnett’s multiple comparison test was used, comparing magnitude of LTP for each drug concentration to vehicle LTP at each post-drug time point. Statistical analyses were performed using Prism 6 (GraphPad Software, USA) statistical software. Values are presented as mean ± SEM, and statistical significance was preset to *P *< .05.

CSD data were analyzed using 1-way ANOVA (only susceptible mice) followed by Tukey post hoc test. Statistical analyses of PCP-induced locomotor activity were performed using 2-way ANOVA followed by Tukey (distance traveled over time) or Dunnett’s post hoc multiple comparison test (total distance traveled for 40 minutes). All rotarod data were analyzed by ANOVA, followed by Tukey post hoc test using StatView (USA). Unless otherwise explicitly stated, data are presented as mean ± SEM.

## Results

### Zelquistinel Is a NMDA Receptor Modulator

**Figure 1.**
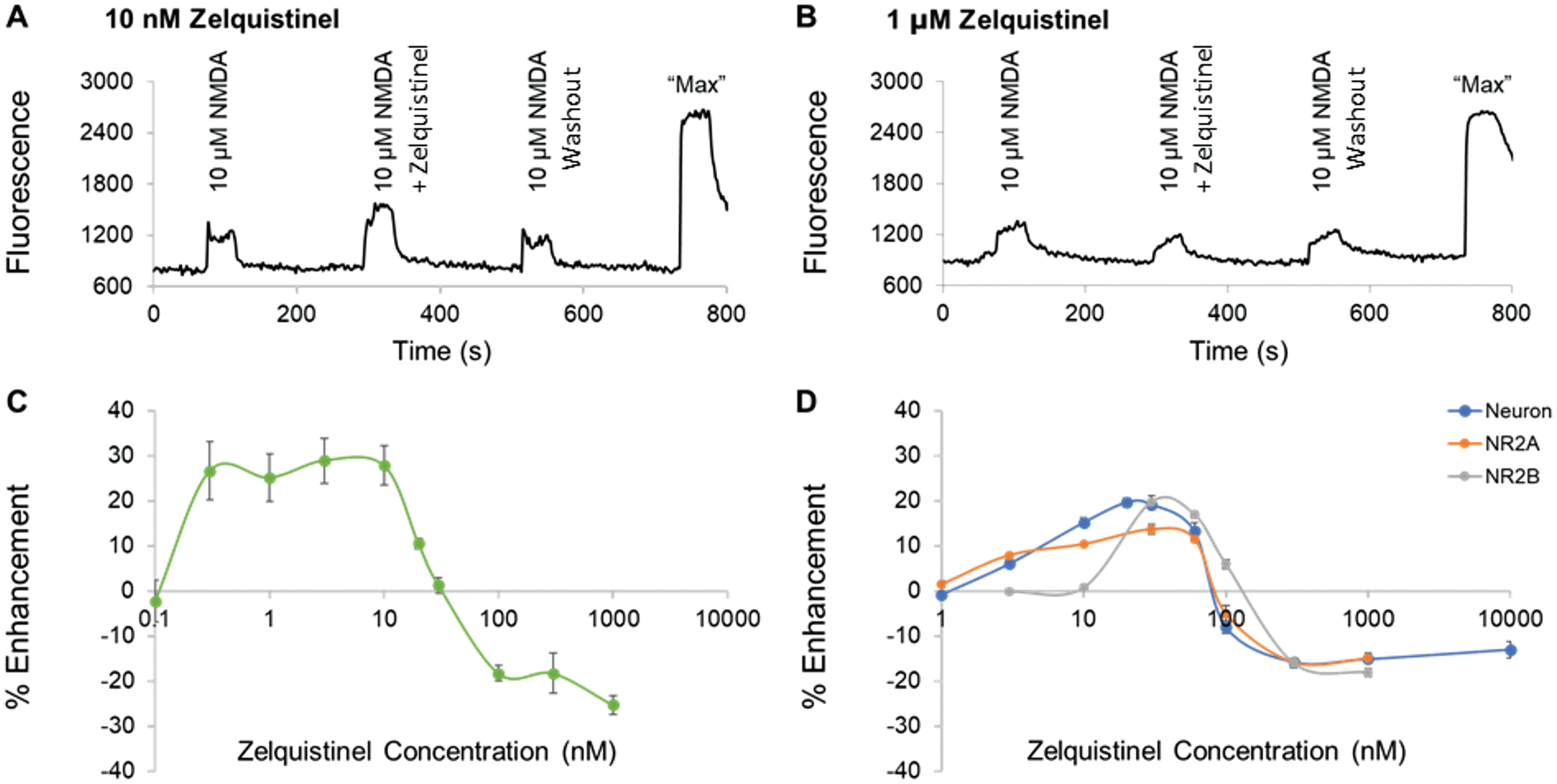
Zelquistinel potentiated NMDA- or glutamate-induced changes in intracellular calcium. Zelquistinel potentiated NMDA-induced intracellular calcium ([Ca^2+^]_i_) changes in rat cortical neurons and in HEK293 cells expressing NR2A or NR2B receptors. Representative traces showing (A) the potentiation effect of 10 nM zelquistinel and (B) the inhibition effect of 1 μM zelquistinel. (C) Dose-dependent effects of zelquistinel on NMDA alone (10 µM) induced [Ca^2+^]_i_ response (n = 4–8 coverslips for each data point). (D) Dose-dependent effects of zelquistinel on [Ca^2+^]_i_ changes in NR2A− (in the presence of 300 nM glutamate + 3 μM D-serine) or NR2B-expressing (in the presence of 100 nM glutamate+3 μM D-serine) HEK cells, or in cortical neurons (in the presence of 3 μM NMDA+3 μM D-serine; neuron) (n = 5–12 coverslips for each data point). Each point (C, D) represents mean ± SEM, and the percent enhancement is normalized to the signal induced by NMDA or glutamate only.

The effect of zelquistinel on NMDAR-dependent intracellular calcium signaling was first assessed in cultured rat cortical neurons. After 3 weeks in culture and in the absence of exogenous D-serine or glycine, NMDA (10 µM) produced a small but significant increase in intracellular calcium ([Ca^2+^]_i_) (Figure 1A–B). While zelquistinel (0.1–1000 nM) alone did not increase intracellular calcium influx, co-application of low concentrations of zelquistinel (0.3–10 nM) with 10 µM NMDA produced approximately 30% potentiation of the NMDA-induced calcium signal, whereas concentrations ≥100 nM partially inhibited (approximately 25%) NMDAR activity (Figure 1C). In the presence of 3 µM D-serine, NMDA (3 µM)-induced [Ca^2+^]_i_ increases were 46.6 ± 1.3% (n = 88 coverslips) of those by 10 µM NMDA. Under these activating conditions, zelquistinel (3–60 nM) potentiated NMDA-induced [Ca^2+^]_i_ and inhibited responses at concentrations ≥100 nM (Figure 1D, neuron). Although the zelquistinel inhibitory concentrations were identical in the absence or presence of D-serine, the zelquistinel-positive modulation range was narrower in the presence of D-serine.

### Zelquistinel Modulated Recombinant NR2A and NR2B Receptors

Zelquistinel’s modulation of NMDAR-dependent [Ca^2+^]_i_ mobilization was also assessed in stable HEK293 cells expressing human NR1-NR2A or NR1-NR2B receptors (Figure 1D). To induce similar levels of receptor activity in the presence of 3 μM D-serine, 300 nM and 100 nM glutamate were added to NR2A- and NR2B-expressing HEK293 cells, respectively, to increase [Ca^2+^]_i_ (NR2A: 43.8 ± 1.9% of “max,” n = 60 coverslips; NR2B: 43.6 ± 2.5% of max, n = 48 coverslips); “maximal” changes in [Ca^2+^]_i_ was defined as the response elicited by 3 μM glutamate+10 μM D-serine. Under these activation conditions, similar concentration-dependent potentiation and inhibition effects of zelquistinel were observed regardless of subtype (Figure 1D).

### Zelquistinel Potentiated NMDAR-Mediated Calcium Influx in Presence of Glycine Site Antagonist MDL 105,519

**Figure 2.**
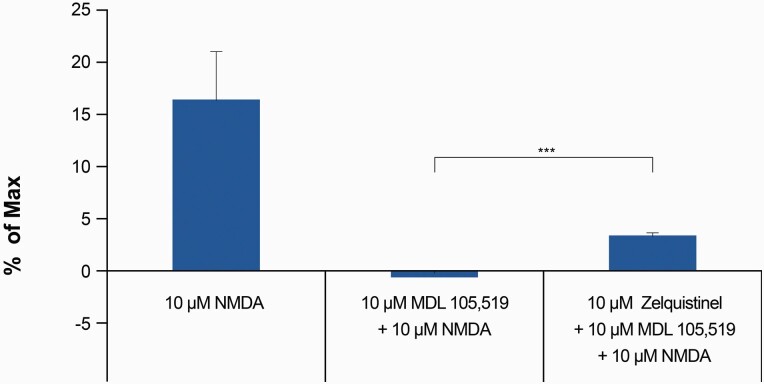
Zelquistinel, co-applied with NMDA, increased intracellular calcium in the presence of MDL 105,519. Effect of 10 μM NMDA, 10 μM NMDA+10 μM MDL 105,519 (MDL), and 10 μM NMDA+10 μM MDL+10 nM zelquistinel on intracellular calcium ([Ca^2+^]_i_). There was an increase in 10 μM NMDA-induced [Ca^2+^]_i_ in the presence of 10 μM MDL when 10 nM zelquistinel was added compared with when zelquistinel was absent (***P < .001). MDL is a competitive glycine site antagonist and completely blocked NMDA-induced [Ca^2+^]_i_ response at 10 μM. Each point represents mean ± SEM (n = 23 coverslips) and is normalized to the signal induced by 10 μM NMDA+3 μM D-serine (Max).

MDL 105,519 (MDL) is a competitive glycine site antagonist (Baron et al., 1996) and completely inhibited the binding of [^3^H]glycine to rat brain membranes with a K_i_ value of 10.9 nM (Baron et al., 1997); 10 μM MDL is an excess, saturating concentration that fully antagonized the glycine co-agonist site. In the presence of 100 μM D-serine, 10 μM MDL also completely blocked the activation of NMDARs by 10 μM NMDA (Donello et al., 2019). In cultured cortical neurons, 10 μM MDL completely abolished NMDA-induced [Ca^2+^]_i_ response (without exogenously added glycine or D-serine), suggesting that low levels of endogenous co-agonist were present (Figure 2). In the presence of 10 μM MDL, addition of 10 nM zelquistinel with 10 μM NMDA induced a significant [Ca^2+^]_i_ increase, demonstrating the ability of zelquistinel to enhance NMDA-mediated NMDAR activation independent of the glycine site (Figure 2). Furthermore, zelquistinel (0.3 nM to 10 μM) does not displace [^3^H]MDL binding from the NMDAR glycine site (percentage displacement <20% for all concentrations; data not shown). In addition, zelquistinel did not exhibit binding affinity for any of the known NMDAR sites, including the agonist site ([^3^H]CGP 39653), PCP site ([^3^H]TCP, [^3^H]MK-801), or polyamine site ([^3^H]Ifenprodil) (data not shown). These data suggest that zelquistinel’s site of action is independent of the glycine co-agonist site or any of the other known modulatory sites within the NMDAR complex.

The pharmacological selectivity of zelquistinel was evaluated in a broad panel of radioligand displacement assays, which included 80 receptors, ion channels, and monamine transporters. The receptor targets included common amino acid and monoamine neurotransmitters receptors, such as γ-aminobutyric acid, glycine, acetylcholine (nicotinine and muscarinic), dopamine, norepinephrine, adrenaline, cannabinoid, opioid, cholecystokinin, endothelin 5-hydroxytyramine, histamine, muscarinic, vasopressin, and steroid nuclear receptors. The ion channels included voltage-gated channels (sodium, potassium, calcium) and membrane ligand-gated channels (NMDA, 5-hydroxytyramine). The monoamine transporters included norepinephrine, dopamine, and serotonin transporters. No significant radioligand displacement was detected at any of the above targets by 10 µM zelquistinel.

### Zelquistinel Enhanced NMDAR-Mediated EPSCs and Facilitated LTP in Rat mPFC Slices

**Figure 3.**
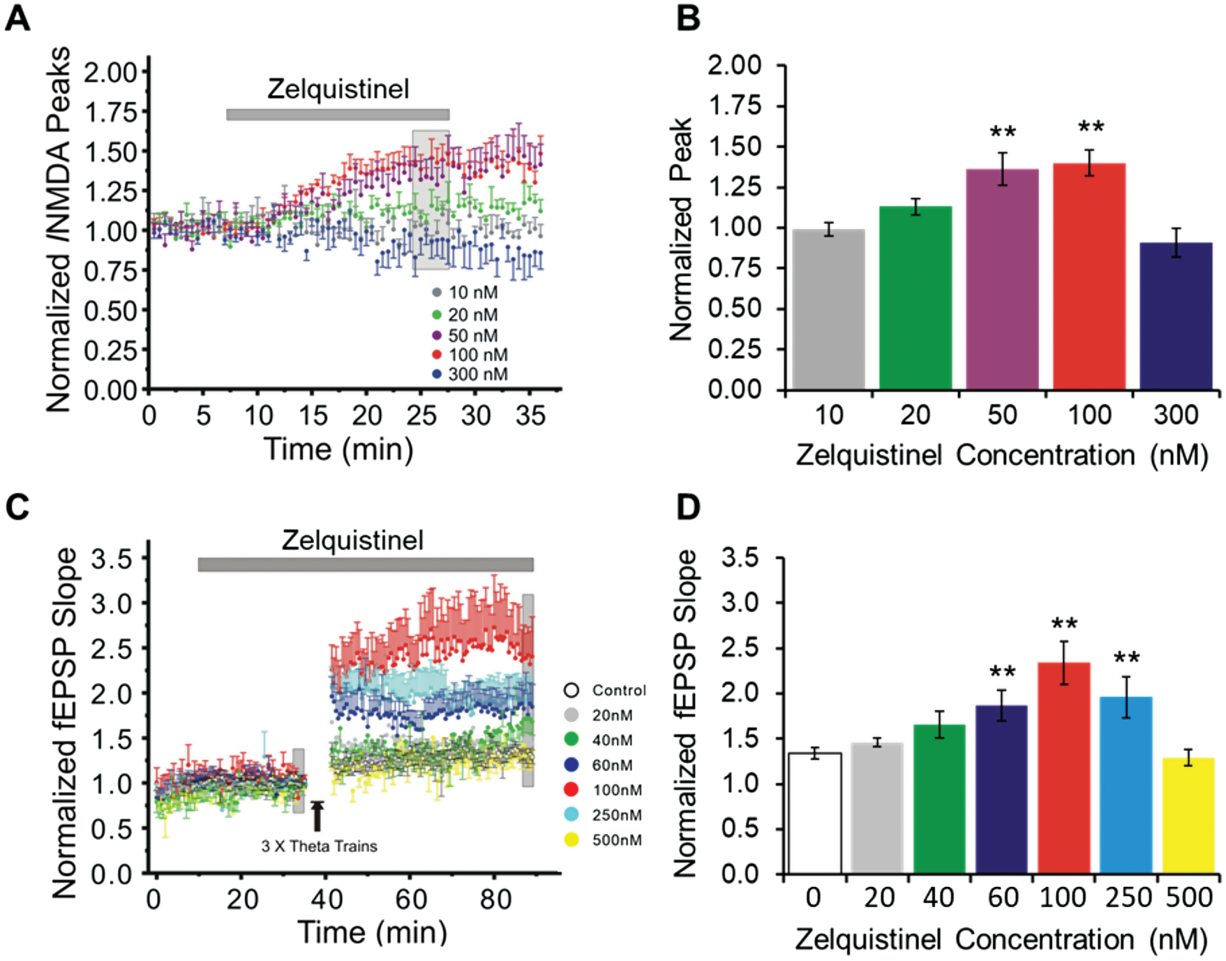
Zelquistinel potentiated NMDAR-mediated excitatory postsynaptic currents and facilitated long-term potentiation in mPFC. (A, B) Zelquistinel potentiated NMDAR-mediated excitatory postsynaptic currents (EPSCs) in mPFC. (A) Time course of experiments comparing pharmacologically isolated NMDAR-mediated EPSCs (20–25 minutes after bath application of drug) with pre-drug baseline. (B) Zelquistinel concentration-dependently increased the magnitude of NMDAR-mediated EPSCs recorded from mPFC pyramidal neurons at 20, 50, and 100 nM concentrations; this effect was not observed at the higher concentration of 300 nM. *P < .01 vs pre-drug baseline in each cell via 1-way ANOVA with Bonferroni correction for multiple comparisons (n = 6–11). (C, D) Zelquistinel enhanced long-term potentiation (LTP) in mPFC. (C) Time course of experiments comparing LTP (of slices pretreated with 20–500 nM zelquistinel) induced by theta burst stimuli (TBS) with untreated slices (control, open circles). (D) Zelquistinel (60–250 nM) enhanced the magnitude of LTP. *P < .01 vs control (control [0 nM zelquistinel]) via Kruskal-Wallis 1-way ANOVA and Kruskal-Wallis multiple comparison Z-values test (n = 6–8 slices for each concentration tested).

Pharmacologically isolated NMDAR-mediated synaptic currents were recorded in layer II/III pyramidal neurons of mPFC via in vitro slices (time course in Figure 3A). Zelquistinel, in a concentration-dependent fashion, significantly increased the magnitude of NMDAR-mediated EPSCs (F_4,39_ = 8.417, *P* < .0001). Multiple comparisons with Bonferroni correction showed that NMDA currents were significantly enhanced by zelquistinel at 50 nM (t = 3.42, *P* = .0059) and 100 nM (t = 3.035, *P* = .0013) (Figure 3B), and there was no potentiation effect at a concentration of 300 nM.

NMDAR-dependent synaptic plasticity (LTP) was evaluated in rat mPFC slices with or without treatment of zelquistinel (0, 20, 40, 60, 100, 250, and 500 nM; time course of LTP induced by TBS in mPFC slices in Figure 3C). Bath application of 60, 100, and 250 nM zelquistinel before and during induction of LTP significantly enhanced the magnitude of LTP in mPFC, whereas 500 nM did not (Figure 3D, Kruskal-Wallis 1-way ANOVA, F_(6,42)_ = 4.7423, *P* = .0049). Kruskal-Wallis multiple comparison Z-values test confirmed that zelquistinel significantly increased LTP in mPFC at 60 nM (z = 2.4154, *P* = .0079), 100 nM (z = 3.3628, *P* = .00039), and 250 nM (z = 2.3649, *P* = .009).

### Zelquistinel Has High Oral Bioavailability and Brain Penetration

Following a single oral or i.v. dose of zelquistinel in male Sprague Dawley rats, plasma, brain, and CSF concentrations and pharmacokinetic parameters were quantified. Zelquistinel’s oral bioavailability was high (approximately 100%), with mean T_max_ at 0.50 hours; mean half-life (t_½_) ranged from 1.21 to 2.06 hours, mean apparent clearance (CL/F) ranged from 9.04 to 11.2 mL/min/kg, and mean apparent steady-state volume of distribution (V_ss_) was 0.57 L/kg (Table 1).

**Table 1.** Pharmacokinetic Parameters^a^ After a Single Dose of Zelquistinel

Dose (route)	T_max_ (h)	t_½_ (h)	CL (mL/min/kg)	V_ss_ (L/kg)	F^b^ (%)
10 mg/kg (PO)	0.50 ± 0.0	2.06 ± 0.66	9.05 ± 0.70	N.C.	~100^c^
2 mg/kg (IV)	—	1.21 ± 0.14	11.2 ± 0.55	0.57 ± 0.06	—

Abbreviations: AUC_last_, area under the concentration-time curve from t = 0 hour to time of last measurable concentration; CL, clearance; F, oral bioavailability; N.C., not calculable; t_½_, half-life; T_max_, time of maximal concentration; V_ss_, volume of distribution at steady state.

Data presented are means ± SD.

^a^Pharmacokinetic parameters are presented as means ± SD.

^b^AUC_last_ considered for the bioavailability calculation.

^c^Due to serial plasma sampling, mean AUC_last_ values were used to estimate bioavailability.

Oral doses of 3 µg/kg to 10 mg/kg produced dose-proportional increases in plasma, CSF, and brain drug exposures (Table 2). At approximately 1 hour after an oral dose of 10 mg/kg, near or at C_max_ concentrations were obtained (mean ± SD [in ng/mL]: plasma, 5991 ± 545; CSF, 1470 ± 775; brain, 1013 ± 560; n = 3 for all; Table 2). Brain to plasma and CSF to plasma ratios were 0.16 and 0.30, respectively.

**Table 2.** Plasma, CSF, and Brain Exposure After a Single Oral Dose of Zelquistinel

	Plasma		CSF		Brain	
Dose	C_max_ (ng/mL)	AUC_last_ (ng·h/mL)	C_max_ (ng/mL)	AUC_last_ (ng·h/mL)	C_max_ (ng/g)	AUC_last_ (ng·h/g)
3 µg/kg	1.3	4.1	0.31	0.84	0.073	N.C.
100 µg/kg	53.8	146	8.39	34.3	3.82	N.C.
10 mg/kg	7300	18450	1470	N.C.	1013	N.C.

Abbreviations: AUC_last_, area under the concentration-time curve from t = 0 hour to time of last measurable concentration; C_max_, maximum observed concentration; CSF, cerebral spinal fluid; N.C., not calculable.

Data presented are means.

### A Single Oral Dose of Zelquistinel Enhanced LTP >1 Week in Hippocampus and mPFC

**Figure 4.**
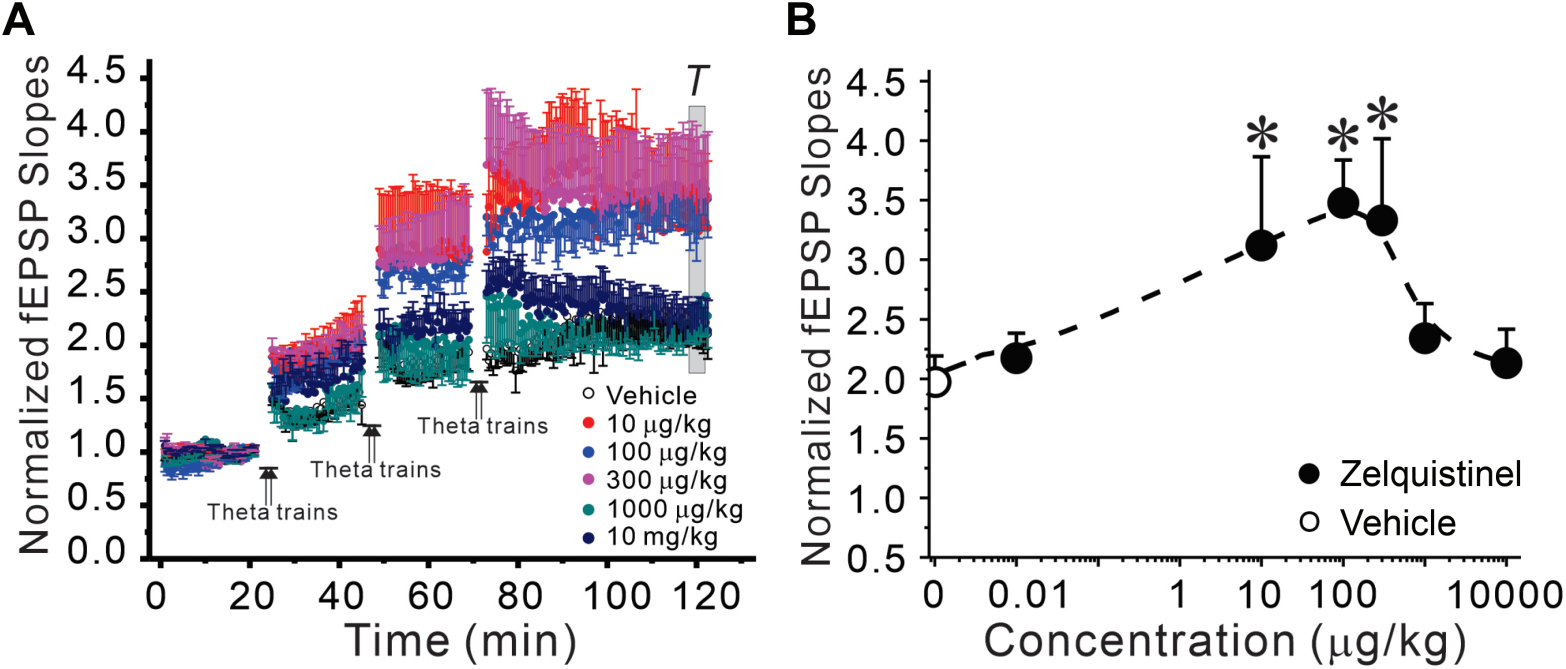
Dose-dependent effect of zelquistinel on metaplasticity in the hippocampus. Long-lasting enhancement of LTP (metaplasticity) in rat hippocampal slices at 24 hours post dose was produced by a single oral dose of zelquistinel. (A) Time course of induction of metaplasticity by zelquistinel (10 µg/kg to 10 mg/kg, PO). (B) Dose-dependent effect of a single oral dose of zelquistinel; the magnitude of LTP, induced 24 hours post dose, is presented as normalized fEPSP slopes after the third and final set of theta burst stimuli, representing maximal LTP at Schaffer collateral-CA1 synapses. *P < .05 vs no-drug control slices via ANOVA followed by Dunnett’s post hoc test comparing each dose to vehicle (n = 10 for each dose tested).

The long-lasting effects of zelquistinel were examined in vitro at Schaffer collateral-CA1 synapses in hippocampal slices 24 hours post dosing when applying multiple TBS stimulus trains at 20 minute intervals. At this timepoint, zelquistinel significantly enhanced the magnitude of LTP (F_6,48_ = 2.921, *P* = .0165) in a dose-dependent manner (Figure 4). Oral doses of 10, 100, and 300 µg/kg all significantly enhanced the magnitude of LTP (Dunnett’s multiple comparison test, *P *< .05 for all; Figure 4B). The 2 highest doses of zelquistinel (1 and 10 mg/kg, PO) did not significantly alter the magnitude of LTP; this provided further confirmation of zelquistinel’s inverted-U dose-response relationship, which was also seen in the in vitro experiments (Figure 3) for enhancement of NMDAR EPSC and LTP.

**Figure 5.**
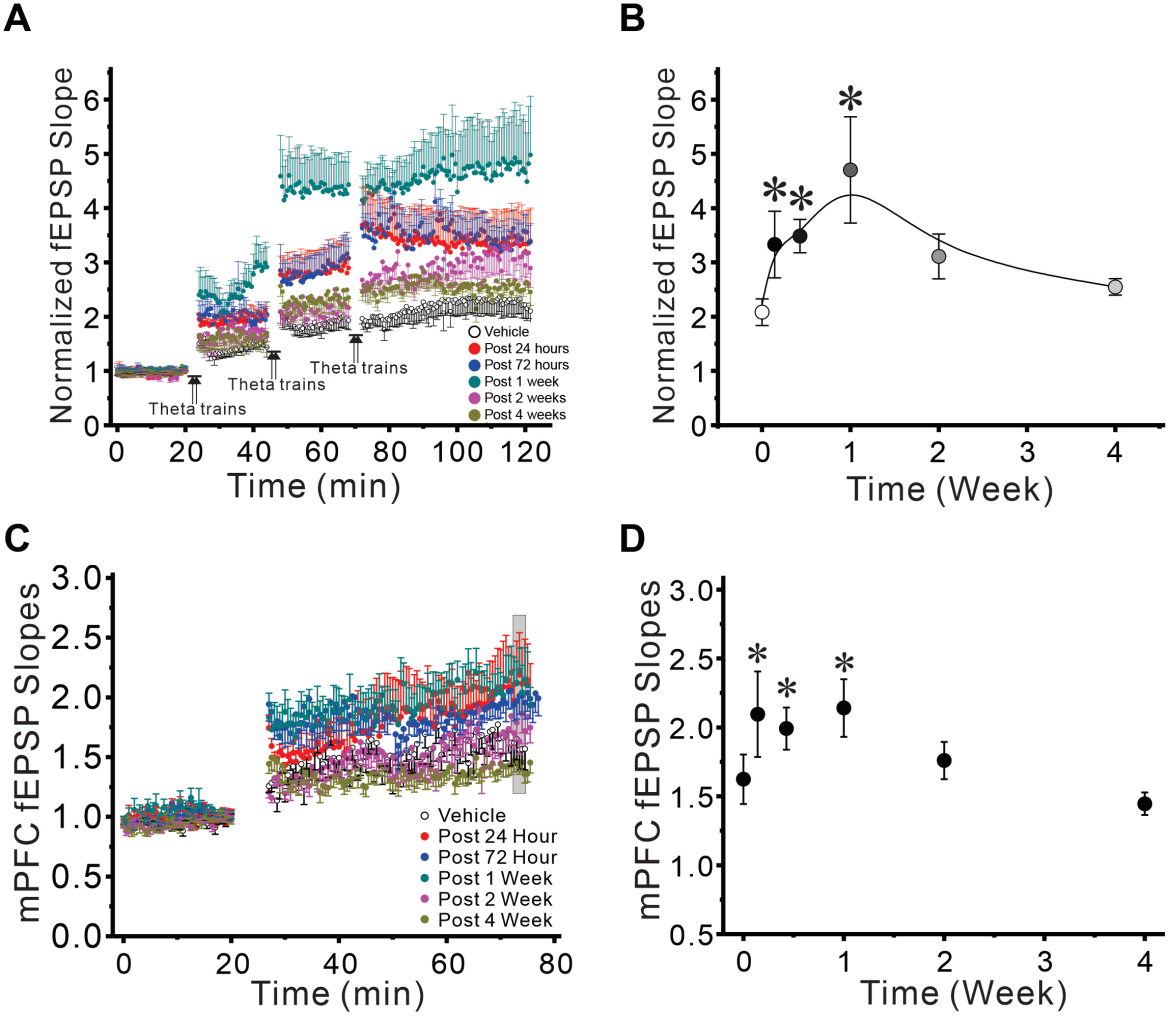
Long-lasting enhancement of LTP induced by a single oral dose of zelquistinel in the rat hippocampus and medial prefrontal cortex. The duration of the metaplasticity effects of zelquistinel in persistently enhancing magnitude of LTP (300 µg/kg, PO) in hippocampus (A, B) and mPFC (C, D). Left panels (A, C): Time course of the effects of zelquistinel on the induction of LTP at 24 hours (n = 8), 72 hours, 1 week, 2 weeks, and 4 weeks post dosing compared with control slices obtained from rats administered vehicle (open circles). Each point is mean ± SEM of stimulus-evoked field excitatory postsynaptic potentials (fEPSP) evoked each 30 seconds. Right panels (B, D): The duration of the long-term effects of a single oral dose (300 μg/kg zelquistinel) on LTP induction in (B) hippocampal and (D) mPFC slices. *P < .05 vs control (vehicle [0 μg/kg zelquistinel]) via ANOVA followed by Dunnett’s post hoc test (n = 6–9). Data point at time = 0 corresponds to vehicle treatment.

The duration of the metaplasticity effects of a single dose of zelquistinel (300 µg/kg, PO) were evaluated in both hippocampus and mPFC. Zelquistinel significantly enhanced the magnitude of LTP at Schaffer collateral-CA1 synapses in the hippocampus up to 2 weeks after administration, with the maximum enhancement observed 1 week post dosing (Figure 5A–B). In mPFC, the peak metaplastic enhancement of LTP was reached at 24 hours post dose and was sustained for 1 week post dosing (Figure 5C–D).

### A Single Oral Dose of Zelquistinel Produced Sustained Antidepressant-Like Effects

**Figure 6.**
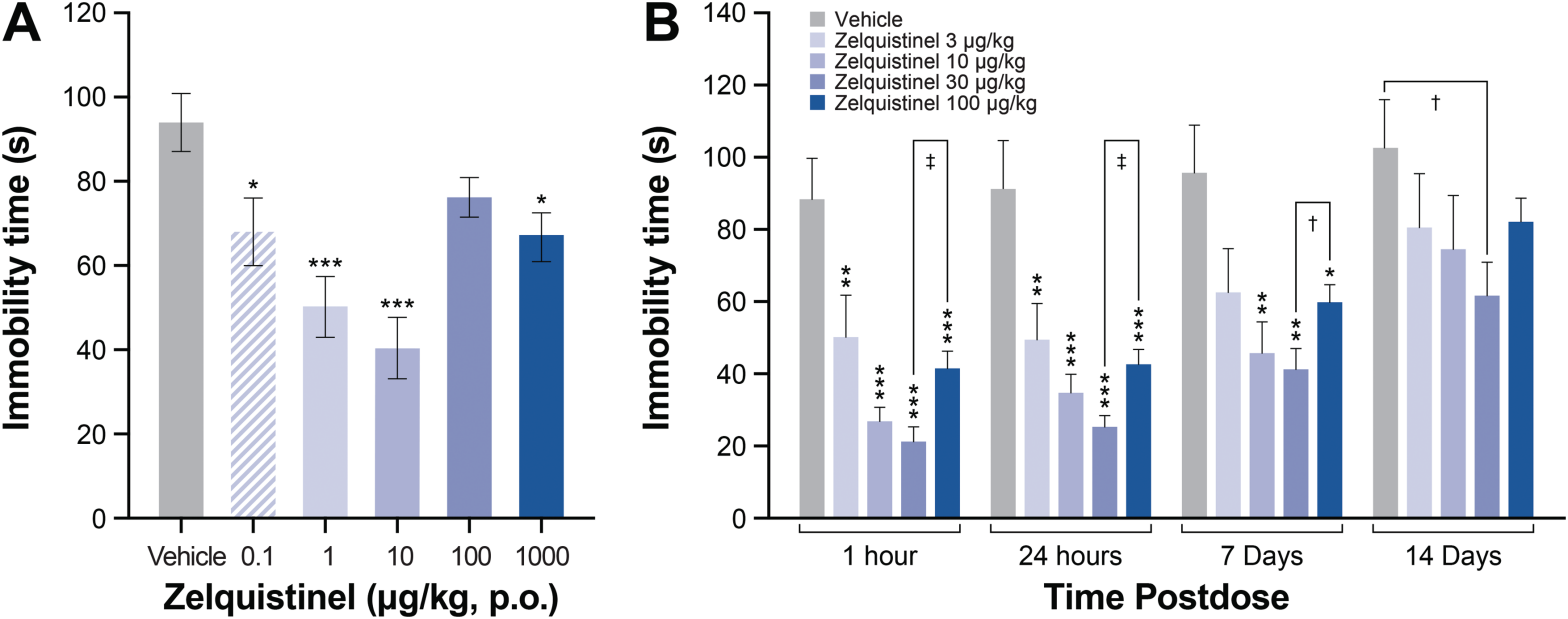
Zelquistinel dose-dependently produced and long-lasting antidepressant-like response. (A) Zelquistinel produced dose-dependent rapid antidepressant-like responses in the rat forced swim test 60 minutes post dose (n = 10 rats for each dose). *P < .05, ***P < .001 vs vehicle via 1-way ANOVA followed by Dunnett´s multiple comparison test. (B) Zelquistinel produces an acute and long-lasting antidepressant-like response with most effective dose of 30 μg/kg (n = 9–10 rats for each dose). *P < .05, **P < .01, ***P < .001 vs vehicle via 1-way ANOVA followed by Dunnett´s multiple comparison test.

In the rat FST, zelquistinel significantly reduced immobility at lower doses (0.1–100 µg/kg, PO) at 1 hour post dose; higher doses were less effective (Figure 6). This U-shaped biphasic dose-response and the sustained efficacy of zelquistinel was confirmed in a long-term follow-up study in rodents, where the antidepressant-like effects of zelquistinel after a single oral dose lasted for >7 days (*P* < .01 for 10–100 µg/kg); the most effective dose was 30 μg/kg (Figure 6B). No sex effects were seen at the 30-µg/kg dose across these same timepoints (n = 6/group; main effect for drug: F_(1,40)_ = 689.8, *P* < .05; time: F_(3,40)_ = 2.0, *P* > .05; sex: F_(1,40)_ = 0.03, *P* > .05; sex × treatment: F_(1,40)_ = 0.3, *P* < .05; sex × time: F_(3,40)_ = 0.01, *P* < .05; time × treatment: F_(3,40)_ = 0.3, *P* < .05; and sex × treatment × time interaction: F_(3,40)_ = 0.3, *P* > .05.

### Rapid Antidepressant Activity of Zelquistinel in CSD Mouse Model

**Figure 7.**
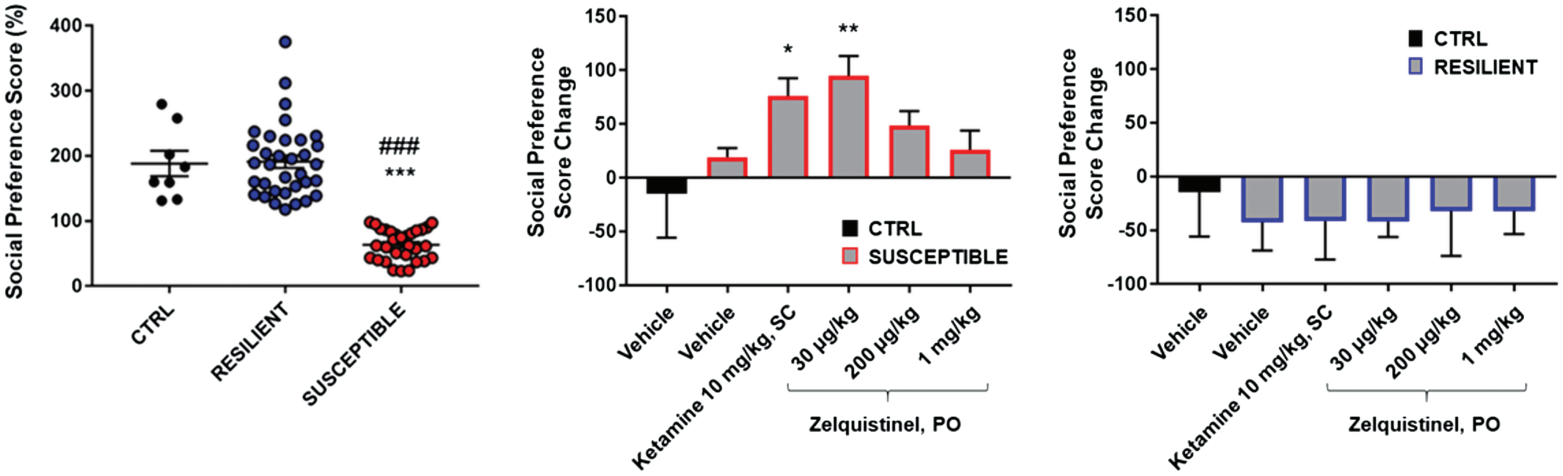
Zelquistinel restored social approach behavior in stress-susceptible mice. (Left) Social preference distribution after chronic social defeat (n = 8–35 per group). ***P < .001 vs control mice (CTRL), ###*P* < .001 vs RESILIENT (stress-resilient mice). Social avoidance phenotype in (middle) stress-susceptible mice (SUSCEPTIBLE) and (right) RESILIENT group (n = 5–8 per group). *P < .05, **P < .01 vs SUSCEPTIBLE+VEH; #*P* < .05, ##*P* < .01 vs SUSCEPTIBLE+30 μg/kg zelquistinel. One-way ANOVA followed by a Tukey post hoc test. All data expressed as mean ± SEM.

The CSD model is potentially a translationally relevant model of depression with respect to both behavioral and molecular endpoints and has been proposed as a key model in antidepressant drug discovery (Berton et al., 2006). Importantly, this model tests for antidepressant-like effects with a different behavioral outcome than the FST (SP vs floating time) and tests for antidepressant-like effects in multiple species (mice vs rats that were used in the Porsolt test). After 10 days of CSD, the social aversion phenotype was seen in 46% of the C57BL/6J mice subjected to the CSD procedure (Figure 7A–B; susceptible mice, *P *< .0001 vs control and resilient groups). Acute treatment with 30 μg/kg (PO) zelquistinel restored social approach behavior as rapidly as 1 hour post administration, and 10 mg/kg ketamine (SC) also restored social approach behavior (Figure 7C). Neither zelquistinel nor ketamine significantly affected the social avoidance phenotype in stress-resilient mice (Figure 7D).

### Inhibition of PCP-Induced Hyperlocomotion

**Figure 8.**
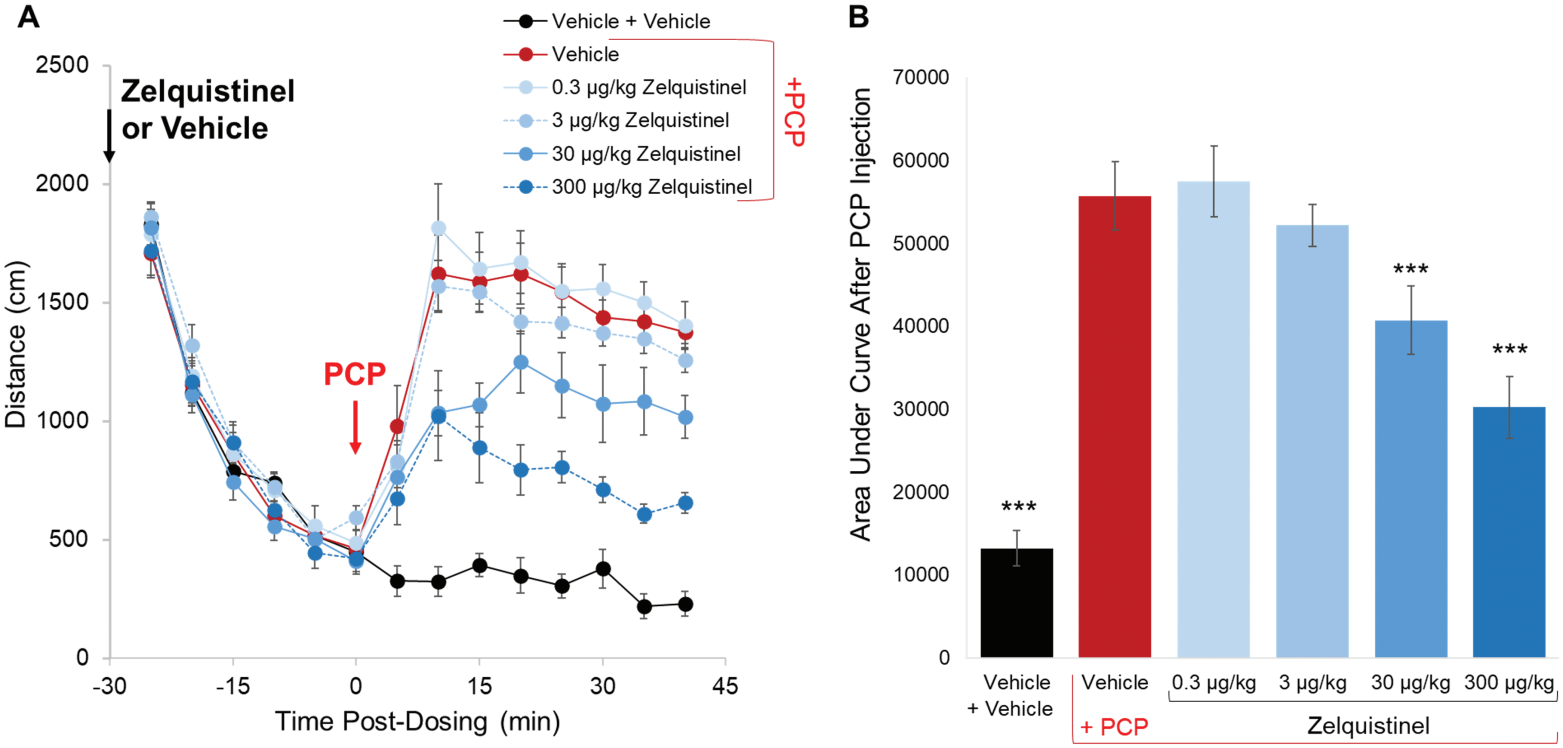
Effects of zelquistinel on PCP-induced hyperlocomotion. Effects of zelquistinel on PCP-induced hyperlocomotion in rats. (A) Time course of the effects of zelquistinel (0.3–300 µg/kg, PO) on PCP-induced hyperlocomotion; time of injection and oral administration of zelquistinel are indicated by vertical arrows. (B) Total distances traveled for 40 minutes after PCP injection. ***P < .001 vs the vehicle/PCP group via 1-way ANOVA followed by Dunnett’s test (n = 12 per group).

PCP is an NMDAR antagonist that produces dose-dependent increases in locomotor activity (Adams and Moghaddam, 1998); the PCP rodent model is sensitive to the activity of antipsychotics and positive NMDAR modulators (Chiusaroli et al., 2010; Alberati et al., 2012). A subcutaneous injection of 2.5 mg/kg PCP significantly increased the locomotion activity in the OF test compared with the vehicle group (Figure 8; red [PCP] vs black [vehicle], *P* < .001 1-way ANOVA followed by Dunnett’s test). Zelquistinel dose-dependently inhibited PCP-induced hyperlocomotion when it was administered 30 minutes prior to PCP injection; the effects were significant at 30 and 300 µg/kg (*P* < .001 vs the vehicle/PCP group via 1-way ANOVA followed by Dunnett’s test). As shown previously, zelquistinel increased synaptic plasticity (300 µg/kg, PO; Figure 5) and produced antidepressant-like effects (30 µg/kg, PO; Figures 6 and 7); these doses also reversed PCP-induced hyperlocomotion, which further provides support that the mechanism of action for zelquistinel is through positive modulation of NMDARs.

### Safety and Tolerability

**Figure 9.**
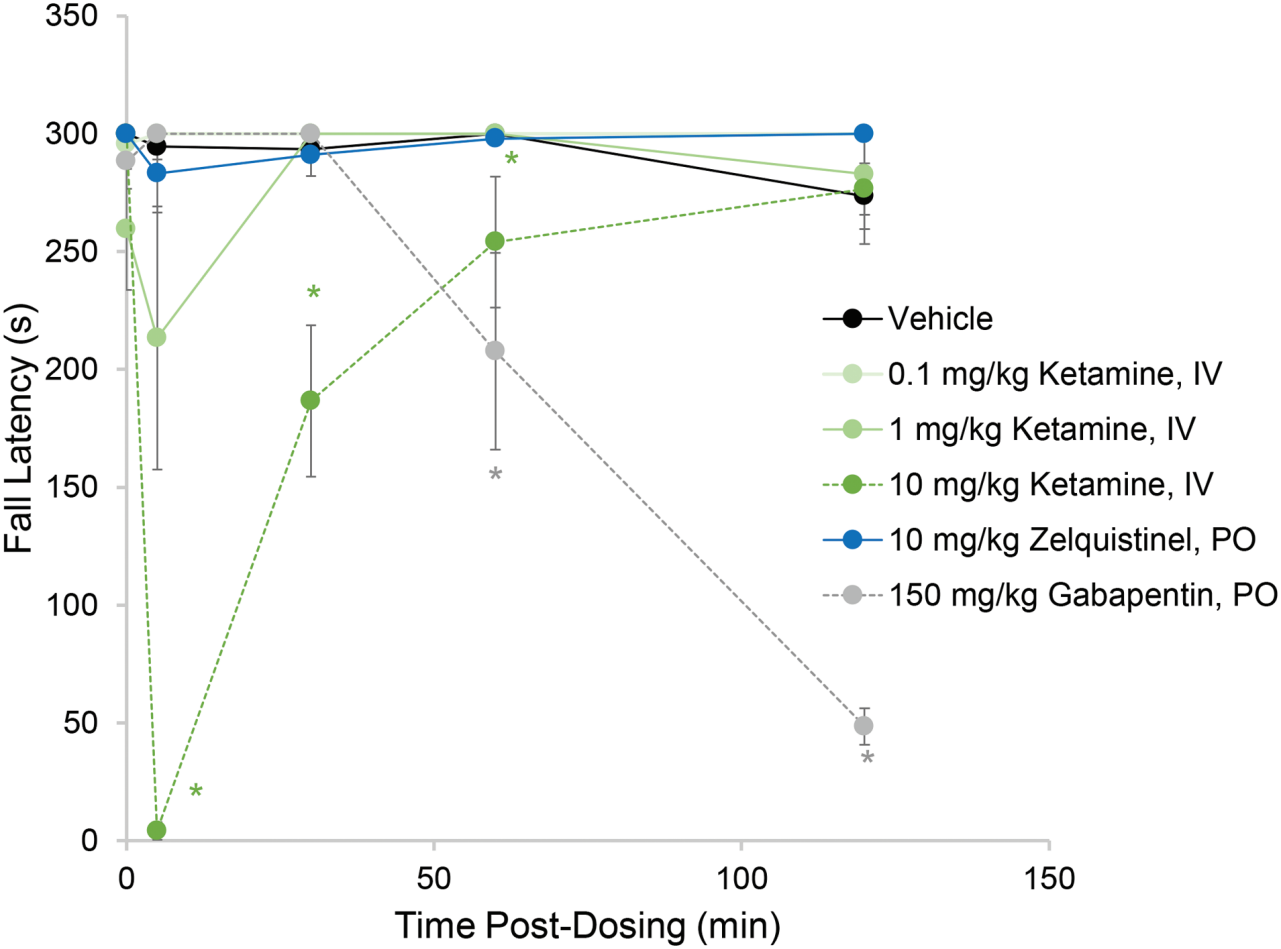
Zelquistinel does not show sedative or ataxic side effects. At 5 minutes before the first test session, rats were dosed with ketamine (0.1, 1, or 10 mg/kg, IV), gabapentin (150 mg/kg, PO), or zelquistinel (10 mg/kg, PO). *P <.05 vs vehicle via a Tukey post hoc test (n = 6–12).

Central nervous system tolerability of zelquistinel was assessed in the rat rotarod test for motor coordination (Figure 9) at a significantly higher dose (10 mg/kg, PO) relative to its antidepressant-like doses (0.1–100 µg/kg, PO). Zelquistinel (10 mg/kg, PO) did not alter fall latencies in the rotarod test relative to vehicle at any of the timepoints tested (5–120 minutes post oral dose). In contrast, ketamine (0.1–10 mg/kg, i.v.) and gabapentin (250 mg/kg, PO) decreased fall latencies either very rapidly (for ketamine) or gradually (for gabapentin) during the 120-minute observation period.

The optimal therapeutic-like dose of zelquistinel (30 µg/kg PO) did not alter fall latencies from 5 to 120 minutes after dosing in both male and female rats (n = 6/group; main effects for drug: F_(1,40)_ = 0.4, *P* > .05; sex: F_(1,40)_ = 0.6, *P* > .05; and time: F_(3,40)_ = 0.3, *P* > .05; and drug × sex: F_(3,40)_ = 0.3, *P* > .05, drug × time: F_(3,40)_ = 0.2, *P* > .05, and drug × sex × time: F_(3,40)_ = 0.1, *P* > .05) interactions. However, the positive control ifenprodil (50 mg/kg SC; female rats n = 6) reduced fall latencies compared with its respective vehicle control group (n = 6 female rats) in a time-dependent manner main effect for drug: F_(1,10)_ = 39.8, *P* < .05, time: F_(3,30)_ = 6.0, *P* < .05, and a drug × time interaction: F_(3,30)_ = 6.1, *P* < .05, with the mean ± SEM fall latencies being 299.2 ± 0.8 across the vehicle timepoints and 296.7 ± 3.3, 219.2 ± 27.7, 159.2 ± 25.6, and 133.3 ± 45.9 at the 5-, 30-, 60-, and 120-minute timepoints, respectively, for the ifenprodil group.

## Discussion

Oral administration of the novel NMDAR modulator zelquistinel (3 μg/kg to 10 mg/kg, PO) demonstrated rapid absorption and clearance and produced dose-dependent exposures in plasma, brain, and CSF. Zelquistinel produced dose-dependent rapid and long-lasting antidepressant-like activity and persistently enhanced long-term activity-dependent synaptic plasticity in both mPFC and hippocampus. In vitro NMDAR-dependent functional assays demonstrated that zelquistinel positively modulated NMDARs at concentrations that enhanced the magnitude of LTP and elicited antidepressant-like effects. Consistent with its modulation of NMDARs, zelquistinel increased NMDAR-mediated synaptic transmission and magnitude of LTP and persistently shifted the threshold in favor of larger LTP (metaplasticity). These results are consistent with a previous report (Pothula et al., 2021) that zelquistinel (AGN- 241751) enhanced NDMAR current and produced antidepressant-like effects and that these effects depended on NR2B containing receptors in excitatory medial prefrontal cortex neurons. Collectively, these results demonstrate that zelquistinel is an oral rapid and long-lasting antidepressant that may have an improved safety profile compared to existing antidepressant drugs.

A series of in vitro and in vivo studies demonstrated that zelquistinel is a highly potent NMDAR modulator that acts independently of the glycine ligand site via a novel binding site within the NMDAR complex. Similar to rapastinel, zelquistinel is an NMDAR allosteric modulator that exhibits a biphasic inverted U dose-response curve (Zhang et al., 2008; Burgdorf et al., 2013; Moskal et al., 2014; Preskorn et al., 2015; Donello et al., 2019). In cortical neuron calcium flux assays, 0.3–30 nM zelquistinel enhanced flux in the presence of D-serine, while a higher concentration range of (3–60 nM) enhanced flux in its absence. Additionally, 3–60 nM zelquistinel enhanced iCa of NR2a-expressing NMDARs, while 10–100 nM zelquistinel enhanced iCa in NR2b-containing NMDARs. In contrast, at even higher concentrations (>100 nM) zelquistinel weakly inhibited (approximately 15%–20%) NMDA or glutamate induced calcium flux across cell types and NMDAR subtypes.

Overall, the efficacy of zelquistinel in the rat FST was well correlated with in vitro NMDAR pharmacology. A single oral dose of zelquistinel resulted in significant antidepressant-like activity in the rat FST, with the most effective dose being 30 µg/kg. At this dose, no sex differences were observed, whereas in preclinical models, sex differences have been observed with ketamine (Franceschelli et al., 2015). Based on linear interpolation from 3 to 100 µg/kg, the CSF C_max_ is estimated to be 2.56 ng/mL (8 nM) after a 30-µg/kg oral dose. Thus, the CSF C_max_ correlates well with zelquistinel’s in vitro enhancement of NMDAR activity. Doses >100 µg/kg were less effective in the FST. Although mouse PK was not performed, a similar dose response was observed in C57Bl6J mice that demonstrated social deficits after 10 days of CSD.

In mPFC pyramidal neurons, zelquistinel increased the amplitude of NMDAR-mediated synaptic currents and enhanced the magnitude of LTP, with peak effects for both at 100 nM. The dose-response relationship in acute slice experiments was right-shifted, perhaps due to tissue penetration of bath-applied drug. When LTP was measured days after oral administration of zelquistinel, the effective dose range was 10 to 300 μg/kg, and there was a week-long duration of enhanced LTP, indicating a long-lasting metaplastic shift facilitating the induction of larger LTP well after the drug was no longer present. Consistent with these findings, transcriptome profiling showed that zelquistinel induced significant mPFC changes within pathways associated with synaptic plasticity 24 hours following a single oral dose of 30 μg/kg (data not shown).

Taken together, these data are consistent with the antidepressant-like effects of zelquistinel. It has been reported that the long-lasting antidepressant-like effects of rapastinel are also associated with a metaplasticity process that persistently enhances LTP in the hippocampus and mPFC (Burgdorf et al., 2015; Vose and Stanton, 2017), similar to the correlation observed for zelquistinel. Taken together, our data suggest that the antidepressant effects of zelquistinel are through its modulation of NMDARs and persistent enhancement of NMDAR-mediated synaptic plasticity. The correlation of zelquistinel plasma concentrations with in vivo efficacy in the rat FST provides a basis for the prediction of human plasma levels in future studies and clinical use.

Zelquistinel does not show hyperexcitability or dissociative effects in dose-escalation studies. Oral administration of 10 mg/kg zelquistinel did not alter fall latencies in the rat rotarod experiments. These results indicate that zelquistinel has minimal risk of hyperexcitability (due to excessive positive NMDAR activation) or sedation/ataxia/disassociation (due to excessive NMDAR blockade). Interestingly, previous reports demonstrated that rapastinel can reverse or block effects of NMDAR antagonists such as ketamine-induced cognitive deficits and MK-801 neurotoxicity (Rajagopal et al., 2016; Vasilescu et al., 2021). Consistent with its positive modulatory activity, zelquistinel also inhibited hyperlocomotion induced by the NMDAR antagonist phencyclidine.

Depression is a chronic, severe, and often life-threatening affliction. The traditional treatments for depression typically take weeks to exert measurable effects and months to achieve full remission of symptoms. In addition to this lag time for treatment response, up to 30% of patients remain depressed despite being treated with multiple, structurally distinct medications (Rush et al., 2006). A new class of antidepressant medication that can act rapidly and effectively would offer significant advantages over the current standard of care (Gould et al., 2019). The antidepressant effects of the NMDAR open channel blocker ketamine have been consistently replicated (Iadarola et al., 2015), but ketamine research in depression has also mostly used intravenous, weight-based approaches for drug administration. Esketamine (intranasal) has been FDA approved for treatment of refractory depression and depressive symptoms in patients with MDD and suicidal ideation or behavior; however, tolerability issues have stopped a recent study of repeated intranasal ketamine due to uncontrolled fluctuation in absorption levels (Galvez et al., 2018). Rapastinel, a tetrapeptide, has a short plasma half-life and low plasma/CNS penetration, limiting its utility as an oral therapeutic. In contrast, zelquistinel demonstrates favorable safety/tolerability and in vivo pharmacokinetic characteristics in both rats and dogs (data not shown) after oral administration. The mean oral bioavailability of zelquistinel in rats is near complete, with favorable brain to plasma and CSF to plasma ratios. Based on these preliminary data, zelquistinel offers distinct advantages over drugs with non-oral routes of administration for the treatment of neuropsychiatric disease. Zelquistinel, unlike rapastinel, is orally bioavailable and has a longer half-life in plasma and CSF, shows a greater therapeutic dose range, and has increased potency compared to rapastinel.

In summary, zelquistinel is a novel NMDAR allosteric modulator that can induce rapid and long-lasting antidepressant-like effects after a single oral administration. The beneficial properties of zelquistinel represent a significantly improved therapeutic profile for the development of therapeutics for the potential treatment of neuropsychiatric diseases associated with alterations in NMDAR-dependent plasticity.

## Interest Statement

J.R. Moskal and J.S. Burgdorf were employees of Aptinyx at the time of the study, and J.S. Burgdorf is currently an employee of Gate Neurosciences, Inc. P.K. Stanton was funded by a grant from Allergan Inc. X.-L. Zhang has nothing to disclose. J.E. Donello was an employee of Allergan at the time of the study.
